# Lived experiences and perspectives of persons with conditions marked by autonomic dysfunction

**DOI:** 10.1371/journal.pone.0355952

**Published:** 2026-08-17

**Authors:** Lisa L. Shah, Patricia A. Kinser, Sara Moyer, Amy Rider, Le Kang, Rachel Decker, Dominik Feliciano, Leslie Lantz, Taylor B. Crouch, Elliot Popoff, Lathika Mohanraj, Joshua W. Hahn, Na Bo, Ron Gharbo, Gisela Chelimsky, James Burch, Amy L. Salisbury, Tom Chelimsky

**Affiliations:** 1 School of Nursing, Virginia Commonwealth University, Richmond, Virginia, United States of America; 2 School of Medicine, Virginia Commonwealth University, Richmond, Virginia, United States of America; 3 School of Public Health, Virginia Commonwealth University, Richmond, Virginia, United States of America; 4 Department of Epidemiology, Virginia Commonwealth University, Richmond, Virginia, United States of America; University of Rijeka Faculty of Health Studies: Sveuciliste u Rijeci Fakultet zdravstvenih studija, CROATIA

## Abstract

**Background:**

Persistent autonomic dysfunction disrupts homeostasis and promotes pathological processes underlying several major chronic conditions including cancer and cardiovascular disease. Little is known about the lived experience of individuals living with conditions marked by autonomic dysfunction.

**Objectives:**

To understand the lived experiences and perspectives of individuals with conditions characterized by autonomic dysfunction.

**Methods:**

A mixed method, cross sectional study was conducted with individuals (n = 489) with conditions marked by autonomic dysfunction, with a focus on recruiting from marginalized populations**.** Participants completed a survey exploring symptoms and experiences with seeking and receiving care for conditions and symptoms marked by autonomic dysfunction. A subset of 45 participants also completed semi-structured interviews.

**Results:**

At the healthcare system level, participants were concerned about provider availability and a lack of support for patients and providers. At the provider level, participants had negative experiences with dismissive providers and positive experiences when providers believed patients. At the patient level, patients reported that anxiety, depression, sleep problems, brain fog, and fatigue were their most common symptoms, and that they often reduced work hours or stopped working. Patients described using their own research more often than provider recommendations to seek wellness treatments.

**Conclusion:**

The lived experience for people with autonomic dysfunction is multilayered, includes the patient’s personal experience, and is influenced by the patient-provider relationship embedded in unsupportive healthcare systems. These experiences may reinforce the physiological dysregulation at the core of many condition and symptoms, potentially exacerbating a feedback loop of distress, invalidation, mistrust, avoidance, and increased vulnerability.

## Introduction

In response to threats, illness, or trauma, the sympathetic branch of the autonomic nervous system launches the fight/flight/freeze response to protect an individual from harm, with simultaneous withdrawal of the parasympathetic system. While such a response is helpful to survival in an acute setting, severe deleterious consequences can manifest when this autonomic dysfunction persists over time. Chronic or persistent autonomic dysfunction involves persistent sympathetic activation (PSA), a physiological state in which the autonomic nervous system is constantly dominated by the sympathetic (fight/flight/freeze) response, while the parasympathetic (rest/digest) response is withdrawn. Autonomic dysfunction disrupts autonomic homeostasis and promotes inflammation and oxidative stress, which are pathological processes underlying major chronic diseases including cancer [1–3], cardiovascular and cerebrovascular disease [4–6], as well as major psychological disorders such as anxiety and depression [7–9]. Individuals experiencing socioeconomic barriers including those experiencing low income, low education, minoritization, and lack of access to medical care are especially susceptible to the impacts of chronic autonomic dysfunction [10]. Despite the major public health implications of autonomic dysfunction, very little is understood about the experiences of these individuals [11,12], which limits the impact of interventions aimed at reducing complications in people with autonomic dysfunction. The purpose of this study was to understand the lived experiences and perspectives of individuals with conditions characterized by autonomic dysfunction. Specifically, we aimed to describe the characteristics of these individuals and factors that impact their lived experience. This study focused on the health needs, perceptions, experiences and barriers to care of marginalized populations (e.g., minoritized, low-income, low-education, rural/medically underserved communities) and other vulnerable communities (e.g., Veterans) with autonomic dysfunction to identify potentially modifiable targets for interventions and to inform future intervention work in this field.

When activated by threat or trauma, the fight/flight/freeze response driven by the sympathetic branch of the autonomic nervous system is initiated through central nervous system connections between the amygdala, the peri-aqueductal gray (PAG) region, the hypothalamus, and their descending networks. Simultaneously, the hypothalamic-pituitary (HPA) axis, a neuroendocrine feedback mechanism, is activated and the parasympathetic branch of the autonomic nervous system withdraws its rest and digest response, reducing vagal tone to permit domination of the sympathetic response. When the parasympathetic response is diminished, a physiological state of emergency operation and resource depletion ensues. Although a powerful and critical life force, constant survival mode or persistent sympathetic activation with parasympathetic withdrawal is inefficient and can manifest as persistent hypervigilance coupled with fatigue. Subsequent long-term reorganization of the autonomic and central nervous system may occur leading to reduced physiological regulation and recovery [13]. Chronic autonomic dysfunction fosters chronic low-grade inflammation with an increased level of proinflammatory cytokines that worsen nearly every physical or emotional disorder/symptom and contribute to chronic illness [14,15]. Autonomic dysfunction, driven by persistent domination of the sympathetic response and withdrawal of the parasympathetic response, and its multi-system disruption of homeostasis is understood as an underlying mechanism shared across many health conditions and symptoms.

Individuals exposed to a higher degree of uncertainty, threat or an inability to control their life circumstances are at highest risk for autonomic dysfunction, and they often have decreased access to trustworthy medical care. Stigma, defined as a negative or dismissive attitude towards another person or group based on race, gender or religion, or towards a characteristic the person has, acts as a chronic stressor on the individual and activates sympathetic activity [16,17]. Racial discrimination produces significant structural brain changes and changes in connectivity that affect the salient, default and executive control mode networks. Many of the brain regions affected by racial discrimination are involved in activating the sympathetic response [15]. Black individuals who have experienced more discrimination have greater activity of the salient network and the amygdala [15]; and structural racism contributes to worse outcomes in diseases including breast cancer treatment [18].

Furthermore, marginalized populations have a documented distrust of the medical community and decreased access to care. For example, Hispanic females have poor bladder health due to poor access to medical care secondary to socioeconomic disadvantages [19]. Underrepresented groups also have difficulties getting medical care for Long-COVID [20]. Misdiagnosis further exacerbates this problem. Even when patients are able to be seen clinically, their symptoms are frequently diagnosed as anxiety, which negates the underlying autonomic dysfunction and leads to growing frustration and despair that reinforces autonomic dysfunction [21–23]. Despite a compelling need to address the health-related adverse consequences of autonomic dysfunction in marginalized populations, few studies have attempted to systematically characterize the factors that perpetuate or exacerbate this condition, or the experiences of these individuals as they relate to interactions with their health care system.

## Methods

This cross-sectional, sequential mixed-methods study focused on understanding participant experiences through a survey and qualitative interviews. Our aim was to understand participant experiences with seeking or receiving healthcare for their symptoms or conditions. We integrated the quantitative and qualitative findings to provide a more complete picture of the participants’ experiences. This research protocol was approved by the Virginia Commonwealth University IRB (HM20028440).

### Reflexivity and positionality statement

In alignment with mixed-methods quality frameworks, we explicitly acknowledge our positionality. The primary analysts (LS, PK, SM, AR, RD, LL, LM, AS) are all nurses and researchers. Our multidisciplinary co-authors including physician scientists (RG, GC, TC), biostatistics (LK, NB), and psychology (TBC) also critically reviewed our study throughout its design, analysis, and interpretation to challenge and enhance our insights and assumptions. Our prior experience working with patients with a range of conditions and symptoms provided valuable contextual insight during the qualitative interview phase but also introduced potential assumptions regarding what type of quantitative analyses were considered. To mitigate potential bias during data integration and analysis, we utilized an audit trail, analyst triangulation, peer debriefing (EP), and frequent discussions with our multi-disciplinary team to ensure that the integrated findings accurately reflect the data.

### Participants

Participant recruitment began 11/18/2023 and ended 6/7/2024. Participants were recruited through local outreach (e.g., active recruitment of patients in the university’s affiliated academic health system through the electronic health record; flyers in local healthcare clinics), community-based strategies (e.g., social media platforms posts; email listservs; flyers posted in community settings such as libraries, bookstores, and shops) and word-of-mouth. Recruitment through the electronic health record was facilitated through an honest broker process, using ICD codes corresponding to one or more conditions related to chronic autonomic dysfunction, including COVID, generalized anxiety disorder, major depressive disorder, post-traumatic stress syndrome (PTSS), mild traumatic brain injury, postural orthostatic tachycardia syndrome (POTS), cancer, cardiovascular conditions, autonomic dysfunction, chronic fatigue, and chronic pain conditions. To target underserved populations, an additional filter of insurance status was used to identify potential participants that were on Medicaid. Email addresses for a random subset of eligible participants were provided to the study team. Interested individuals were sent a link to the survey landing page on REDCap.

Participants were eligible if they self-identified as having current health concerns and/or symptoms relating to autonomic dysfunction, with or without a diagnosis by a healthcare provider, and were 18 years of age or older. For example, IRB-approved recruitment materials used language such as: “We want to hear your experiences! Have you experienced any of the following symptoms or diagnoses? - chronic or unexplained fatigue, brain fog, chronic pain, depression, anxiety, mood changes, PTSS, fast heartbeat, POTS, cancer, long-COVID, heart concerns, lung concerns, belly concerns, traumatic brain injury, or concussion.” Participants provided electronic consent to participate in the survey. At completion of the survey, participants indicated whether they would be interested in a follow-up interview about their experiences. Prior to beginning the one-on-one semi-structured interview, a member of the research team reviewed and verified consent verbally with each participant. Participants did not have to agree to an interview in order to complete the survey. Participants who completed the survey could opt to enter a raffle for a chance to win $50 e-gift cards; those who completed the interview received a $50 e-gift card.

### Data collection

#### Survey.

A link to the online 15-minute REDCap survey was provided to potential participants through recruitment materials, including emails, newsletters, and QR codes on flyers distributed in the community. The survey began with eligibility screening and consent processes. Eligible participants who consented to the survey were automatically directed to the survey. The investigator developed survey gathered information about participant characteristics and health conditions, symptoms, including the validated Fatigue Severity Scale [24], non-pharmacological treatments, impacts on employment or schooling, and experiences with healthcare providers and the healthcare system.

#### Interview.

A one-on-one semi-structured interview was conducted by one of two interviewers (AR, DF), in a subset of participants who completed the survey and self-identified as willing to participate in an interview. Email invitations were sent to interested individuals, with targeted sampling of members of underserved populations (e.g., Medicaid, non-white) to be demographically representative of the survey sample. The interview was held electronically (via Zoom), lasted approximately 60 minutes and began by inviting the participants to provide some personal history, “Tell me a little about yourself and a bit about why you signed up for the study?” The interview continued with participants being asked to share their experiences with their health condition or symptoms. Specific prompts included “In what ways have your symptoms impacted your life?”, “How have aspects of your identity influenced your experiences of seeking healthcare for your symptoms/condition?”, “Describe your experiences seeking healthcare to treat these symptoms?”, “Where have you turned for information or support with your symptoms?”, “Describe what, if any, treatment you have received for your conditions.”, and “How has your condition/symptoms impacted your emotional health and well-being?”. The interviewer invited participants to share what they wish people knew about their experience, what questions they think are important to ask, and what they have learned from their experiences. Real time member checking was used to verify our understanding during the interviews. The interviews were tested amongst team members prior to implementation and then conducted and audio recorded online via Zoom, and transcribed verbatim, including any fieldnotes/observations taken by interviews during or after interviews.

### Data analyses

Survey data was exported from REDCap into SAS version 9.4 for analysis. Descriptive statistics were calculated for survey variables including means and standard deviations (or median and range) for continuous variables, and frequency counts and percentages for categorical data. Chi-squared, Fisher’s exact, or T-tests were used to compare the participants who interviewed to the participants who only completed the survey. Each variable was analyzed using non-missing records; the number of non-missing records for each is listed in each table.

A qualitative descriptive approach was used for the interview data [25], with analyses conducted via thematic analysis [26]. To begin, three study team members (AR, PK, LS) met to discuss potential biases and agree upon processes. The team acknowledged that two members have personal experiences with hard-to-treat conditions and the team agreed to maintain awareness and continuous discussions of how these experiences could potentially influence interpretation of findings. They began the analysis process by reviewing each transcript in its entirety to gain a sense of the data as a whole. Then, the qualitative analysis team independently reviewed a subset of the transcripts, highlighting quotes and making preliminary coding notes, and subsequently met to discuss the preliminary coding and develop a draft codebook. In the next round of coding, each analyst reviewed eight uncoded transcripts using the draft codebook, noting changes to be made to the codebook. At the next meeting, the team discussed and revised the codebook. In the final round of coding, each reviewer used the revised codebook to code 16 transcripts. A fourth member of the qualitative analysis team (EP) read a random subset of the transcripts to agree or disagree with the coding, serving as a peer debriefer [27]. Finally, the entire group then met to discuss and decide upon final themes. In alignment with best practices [28], several methods to ensure trustworthiness of the qualitative analysis process were used in this study. These methods included maintaining an audit trail for confirmability; utilizing a peer debriefer for credibility; and, maintaining robust discussions among analysts (analyst triangulation) for credibility [27]. Data saturation was achieved as no new codes were derived during the final round of coding.

The qualitative analysis was completed independently of the quantitative analysis before integration of both. The quantitative results are presented with corresponding qualitative results to provide deeper understanding of the lived experiences and perspectives of people living with conditions marked by autonomic dysfunction. A joint display was developed to visualize the qualitative themes and exemplar quotes alongside corresponding quantitative data. Integrative summaries were developed to evaluate findings from a mixed-method perspective.

## Results

A total of 489 participants completed the survey and 45 of these also completed a qualitative interview. All participants experienced manifestations associated with autonomic dysfunction, defined as reporting at least one of the following conditions: chronic or unexplained fatigue, brain fog, chronic pain, depression, anxiety, mood changes, PTSS, fast heartbeat, POTS, cancer, long-COVID, heart concerns, lung concerns, belly concerns, traumatic brain injury, or concussion. Among the 375 participants interested in an interview, 142 were invited to schedule an interview, 63 interviews were scheduled with 45 interviews completed. Participants who completed the interview were older (*M* = 43.4, *SD* = 15.3) than the participants who only completed the survey (*M* = 36.9, *SD* = 13.3), *t*(472) = −3.05, *p* = .003; however, there were no other significant differences between the groups (Table 1). Minimal data was missing and determined to be random with non-significant influence on findings.

**Table 1:** Participant Characteristics.

Characteristic	Participant Group N = 489	
	Survey Only (*n = 444*)	Interview & Survey (*n = 45*)
	*n* (% of Group)	*n* (% of Group)
Sex (*N* = 489)	*n = 444*	*n = 45*
Male (*n* = 137)	129 (29)	8 (18)
Female (*n* = 352)	315 (71)	37 (82)
Gender (*N* = 472)	*n = 429*	*n = 43*
Cisgender (*n* = 424)	385 (90)	39 (91)
Transgender (*n* = 33)	30 (7)	3 (7)
Other gender identities (*n* = 15)	14 (3)	1 (2)
Race (*N* = 480)	*n = 436*	*n = 44*
Black or African American (*n* = 201)	183 (42)	18 (41)
Native American/Alaska Native (*n* = 11)	11 (3)	0 (0)
Native Hawaiian/Pacific Islander (*n* = 3)	3 (1)	0 (0)
Asian (*n* = 11)	8 (2)	3 (7)
White (*n* = 227)	208 (48)	19 (43)
More than one race (*n* = 16)	15 (3)	1 (2)
Other (*n* = 11)	8 (2)	3 (7)
Insurance Status (n = 487)	*n = 442*	*n = 45*
Yes, plan through you or spouse employer (*n* = 151)	140 (32)	11 (24)
Yes, plan you purchased yourself (*n* = 63)	60 (14)	3 (7)
Yes, Medicare, Medicaid, government program (*n* = 212)	186 (42)	26 (58)
Yes, other including COBRA (*n* = 13)	11 (2)	2 (4)
Yes, but not sure what type (*n* = 21)	20 (5)	1 (2)
No insurance (*n* = 27)	25 (6)	2 (4)
Education (*N* = 489)	*n = 444*	*n = 45*
Some high school (*n* = 10)	10 (2)	0 (0)
High school diploma (*n* = 51)	48 (11)	3 (7)
Some college (*n* = 104)	93 (21)	11 (24)
Associates or certificate program (*n* = 75)	68 (15)	7 (16)
Bachelor’s degree (*n* = 169)	154 (35)	15 (33)
Graduate degree (*n* = 80)	71 (16)	9 (20)

**Fig 1:**
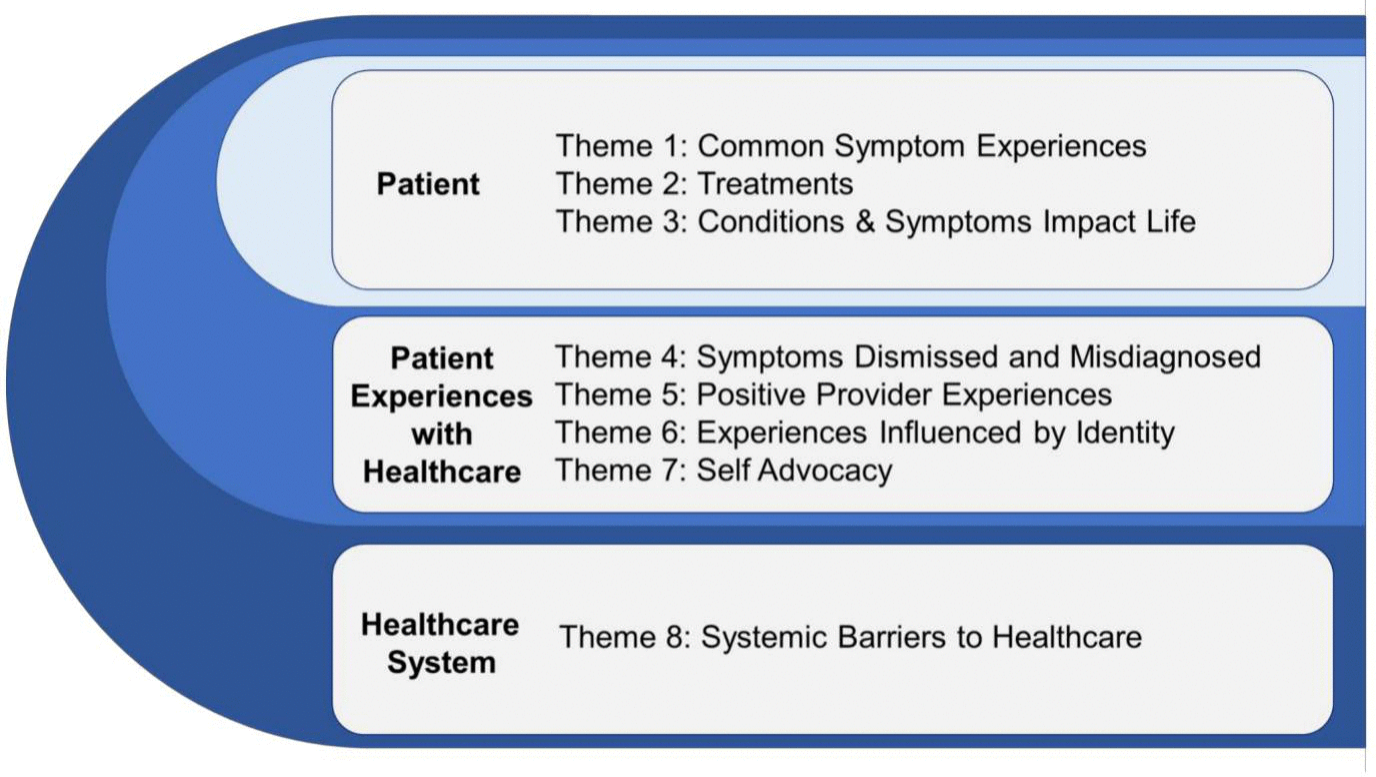
Schematic of qualitative findings. Represents the three levels of participant’s experiences identified through thematic qualitative analysis. Themes are organized based on which level the theme encompasses (Patient, Patient Experiences with Healthcare, and Healthcare System). Although an inductive approach was used, the resulting themes aligned with the socio-ecological model [30].

Thematic analysis of the qualitative data revealed eight themes embedded in three key layers of participants’ experiences: at the level of the healthcare system, at the level of direct interactions with healthcare providers, and at the personal patient-level experience (Fig 1). At the patient level, participants discussed their common symptom experiences (Theme 1) and a wide range of non-pharmacological treatments (Theme 2) they used to address their symptoms. They also described the impact of their condition on daily life (Theme 3) outside of the healthcare system including significant financial barriers and challenges obtaining and using medical accommodations. The patients’ experiences with healthcare were reflected at the interpersonal level through direct interactions with healthcare providers, including negative interactions marked by dismissal or misdiagnosis of symptoms (Theme 4) and positive experiences (Theme 5). These interpersonal experiences were found to be influenced by personal identities of the patient and the healthcare providers (Theme 6). Self-advocacy (Theme 7) was used by patients to navigate these interpersonal relationships. Participant experiences were also reflected at the healthcare system organizational level, discussing broader and systemic barriers to healthcare (Theme 8) including key concepts related to community and public policy such as seeking care within a larger healthcare system. Our quantitative findings are presented below, accompanied by corresponding qualitative findings with anonymized participant quotations.

### Patient

#### Theme 1: Common symptom experiences.

Depression, anxiety, sleep problems, brain fog, difficulty concentrating and fatigue were the most common symptoms experienced in study participants. Participants frequently reported experiencing multiple symptoms, but most frequently identified depression, fatigue, and anxiety as their most concerning symptom (Table 2).

**Table 2:** Participant Symptoms.

Symptom Experienced by Participant	Experienced at All n (% of Group)	Most Concerning n (% of Group)
	*(n = 485)*	*(n = 489)*
Depression	317 (65)	84 (17)
Anxiety	316 (65)	74 (15)
Sleep problems	272 (56)	20 (4)
Forgetfulness or memory problems/ brain fog	259 (53)	32 (7)
Difficulty concentrating	260 (53)	30 (6)
Ongoing fatigue	269 (45)	82 (17)
Getting tired quickly with exercise	201 (41)	16 (3)
Stomach or bowel problems	194 (40)	21 (4)
Ongoing joint or muscle pain	186 (38)	45 (9)
Fast beating or pounding heart/ heart palpitations	164 (34)	9 (2)
Dizziness on standing	157 (32)	10 (2)
Difficulty breathing or shortness of breath	154 (31)	18 (4)
Chest pain	137 (28)	13 (3)
Vision problems	110 (22)	4 (1)
Menstrual changes	103 (21)	5 (1)
Sexual problems	85 (17)	6 (1)
Bladder problems	66 (14)	6 (1)
Changes to taste or smell	53 (11)	1 (0)

Despite depression and anxiety being the most commonly reported symptom and the most concerning symptoms among participants, fatigue and brain fog were the most salient symptoms and poorly understood symptoms. The findings indicate that profound fatigue has a detrimental impact on participants’ cognitive abilities and activities of daily living, “*If I get up and I fix a real good breakfast. I gotta rest before I eat it... I do one thing a day, one thing per day*” *[Participant 325]* and *“it’s like hard to move around in the morning. And so it takes about half of the day to get ready.” [Participant 331].* A college student described the following experience:

*“Ever since having COVID, I feel like a very different person… There is a significant amount of brain fog…it makes it really hard to do things… I feel like I don’t remember things as clearly anymore. It takes longer for me to understand certain things. I find myself spacing out a lot so sometimes I’ll be fully having a conversation with someone and I just go blank. And then I just zone out and…people snap their fingers in front of me to wake me back up again.” [Participant 170]*

#### Theme 2: Treatments.

To help alleviate their symptoms, participants reported using or trying a wide variety of non-pharmacological treatments. The most common treatments included mental health therapy and wellness activities such as physical activity/exercise. Table 3 details the types of treatments that participants tried to address their most concerning symptom.

**Table 3:** Non-Pharmacological Treatments Used for Most Concerning Symptom.

	Tried by Participant n (%)
**Non-Pharmacological Treatment**	*n = 431*
Physical activity/ Exercise	280 (65)
Mental health therapy	235 (54)
Meditation	162 (38)
Diet change	153 (36)
Breathing practices	150 (35)
Prayer	140 (32)
Yoga	100 (23)
Herbal therapies or supplements	98 (23)
Other non-medication-based treatments	50 (11)
Heart rate variability biofeedback	45 (10)
Chiropractor	36 (8)
Acupuncture	27 (6)
Other moving meditation practices (e.g., tai chi, qigong)	25 (6)

Participants described helpful holistic wellness activities, such as physical activity/exercise, meditation, yoga, and acupuncture; with consistency and longer duration optimizing their effectiveness. Building a regular wellness habit proved to be highly beneficial:

“*I feel like for me it was built over time... walking in nature or anything that seemingly is very mundane but realizing that bringing attention into just living in the present moment and, recognizing that this is my life. It sounds so simple, but the feeling of a weight being lifted when recognizing how empowering it is to be able to create the reality that I would want long term through my present actions… this one time specifically I was really struggling with depression for a long time and I remember waking up and literally feeling so light.” [Participant 122]*

Persistent, distressing symptoms prompted participants to explore alternative options. For example, a participant discussed that they “*started to turn to the holistic side of medicine… I am willing to try anything. Acupuncture… took away a lot of my chronic pain, it calmed me down and soothed my stress… My neurologist said ‘it’s good that you’re being your own advocate’.”[Participant 263]* Many participants described the importance of the patient/therapist relationship and having a witness to the struggles even in the absence of tangible solutions. Participant 40 stated that *“I personally really love therapy, so whenever I can talk to somebody about whatever is going on, it just kinda helps like unload, even if like, you know, they’re not there to solve your issues.”* Another participant in therapy developed new coping techniques: *“*

*“Today I had therapy and we discussed flashbacks and addressing ruminating and we worked on some grounding techniques … In terms of mental health, what’s made a big impact [is] just recognizing that I was fairly sad for a long time and I just wanted more to live for.” [Participant 122]*

Conversely, some participants highlighted that not all mental health support providers are a good match: *“I think I went through 4 or 5 different people [counselors] before I found somebody that I felt really understood what I was going through.” [Participant 115]*

When using non-pharmacological treatments, a majority (56%) of patients followed their healthcare providers recommendations; however, 15% did not discuss their treatment with their provider and 6% used a treatment against the advice of their healthcare provider. A majority (81%) of participants reported doing their own research about potential treatments. Likewise, during the interviews participants discussed how helpful it was to learn new information and share it with their support system. For example, one participant described that they *love information…So anything that I feel like I’m learning from, I try- a book, articles – absorbing information, discovering a new thing – that helps me out tremendously. Watching movies and talking on the phone with friends and family also helps me out a lot.” [Participant 262]*

#### Theme 3: Conditions and symptoms impact life.

Even with treatment, most participants’ symptoms had major impacts across many aspects of their lives in terms of their employment or schooling; 19% had lost or changed their job or had to leave school due to their condition or symptoms (n = 77), 42% had to reduce their work or school hours (n = 165). During the interviews, participants discussed the financial barriers and the challenges accommodations associated with their condition. It should be noted that participants that completed interviews were more likely to have lost or changed their job or left school due to their condition than those who only completed the survey, who were more likely to reduce their hours (*X*^*2*^(2, *N =* 409) *=*11.27, *p* *= .0004)*.

Participants expressed concerns regarding unstable employment resulting in financial stress. These challenges were further compounded by the difficulty of finding employers willing to provide accommodations for chronic illnesses For example, one participant described that *“my voice impairment has really taken a toll on my work. I lost my full-time job and now I have to work two part-time jobs to equal a full-time job…I have to find a career or job that is allowing me to or that- that’s willing to accommodate my disability.” [Participant 336]* Further, job instability and healthcare costs contributed to depression and anxiety. This is elucidated in one participant’s statement as follows:

*“I feel like there is definitely a level of depression and I’m definitely anxious. I think financially it’s costly and that’s impacted me a lot too, which also contributes to thinking about [medical equipment] that was gonna be $600 out of your paycheck when you get paid every 2 to 3 weeks or twice a month.” [Participant 124]*

Additionally, participants described multiple negative experiences related to their necessary accommodations at school, work, and in public. Participants described providers’ refusal to sign accommodation paperwork; one participant recalled that their doctor, *“wasn’t going to sign any of my paperwork for accommodations because he ‘didn’t think I was bad enough to need accommodations for anything’,” [Participant 54]* and recalling that coworkers were *“annoyed that I was like getting to work remote and doing all these like special things in their eyes.” [Participant 94]* Several participants described the stigma and negativity they received from both strangers and people they knew related to their use of these necessary accommodations. For example, *“there have always been issues with the service dog, even at the hospital” [Participant 581]* and highlighting that using visible accommodations in public spaces often provokes unwanted attention, questions, and challenges as evidenced by this participant’s story: *“I definitely get stares in public, I get asked very often like questions like: ‘What’s wrong with you?’”* adding that “*I think I also get extra stares because I’m young, people just don’t expect a young person to need a mobility aid. I have been told, ‘Oh, you’re so young’! It’s like, yeah, I know that.” [Participant 359]* These struggles often necessitated the pursuit of legal action due to an inability to obtain accommodations by other means, *“…so it was nice to have legal documentation of this having nothing to do with special treatment or anything.” [Participant 94]* Outside of work and school, participants faced challenges specific to attending healthcare visits with their service animals and obtaining medical equipment such as mobility aids, adding an additional layer of complexity to using accommodations even after the struggle to obtain them. For example, one participant shared,

*“It’s hit or miss. I’ll be honest…Some of the places are great. And it all depends on the nurse even taking [service dog] with me for the MRI… [Sometimes they say,] ‘We can’t do this’. I have a portable, collapsible crate for her, I understand she can’t go on with me with a machine-, I get that. She’s going to be in the crate waiting for me after recovery.” [Participant 581]*

### Patient experiences with healthcare

Many (76%) participants had seen a healthcare provider to address their most concerning symptom at various types of clinics (Table 4). Patient-provider interactions were a primary way in which patients experienced the broader healthcare system. These interactions were shaped by symptom dismissal, misdiagnosis, delayed diagnoses, and the experience of superficial evaluation or just “checking boxes”. Still, survey results showed that participants had, on average, more positive interactions with healthcare providers, most notably listening carefully to their concerns and explaining information in a language they could understand. Less positive experiences with healthcare providers were related to skepticism and symptom dismissal. Additionally, only 58% of participants were satisfied with the care they received (Table 4).

**Table 4:** Survey Responses about Experiences with Healthcare Providers.

Type of healthcare provider who manages the majority of healthcare related to top concern:	n = 484		
Family practice/general practice/internist	152 (42)		
Specialist (cardiologist, pulmonologist, etc.) not at a clinic that specializes in treating your top concern.	86 (24)		
Specialist (cardiologist, pulmonologist, etc.) at a clinic that specializes in treating your top concern.	110 (30)		
Other	15 (4)		
**Experience with Healthcare Provider**	Disagree or Strongly Disagree	Neutral	Agree or Strongly Agree
My healthcare provider…	n (%)	n (%)	n (%)
listened carefully to my concerns.	30 (8)	53 (14)	285 (78)
was dismissive of my symptoms.	205 (56)	67 (18)	93 (26)
was skeptical of my symptoms.	190 (52)	71 (19)	105 (29)
helped me create a plan of action to address my symptoms.	55 (15)	70 (19)	240 (66)
was reassuring.	50 (14)	74 (20)	239 (66)
explained information in language I could understand.	18 (5)	58 (16)	287 (79)
I felt…			
judged by my healthcare provider.	214 (59)	70 (19)	80 (22)
my healthcare provider validated my symptoms.	52 (14)	92 (25)	218 (60)
I was satisfied with the level of care I received from my healthcare provider for my symptoms.	62 (17)	90 (25)	213 (58)
My fears about my symptoms were heard by my healthcare provider.	46 (13)	74 (20)	244 (67)

#### Theme 4: Symptoms dismissed and misdiagnosed.

Interviews elaborated on participants’ negative experiences. Healthcare provider symptom dismissal and misdiagnosis resulted in participants being *“nervous about telling [providers about their symptoms] because sometimes I worry that like I won’t be taken seriously”,* being discounted and repeatedly told *‘you’re fine’,* or being hesitant to share their own perspectives *“because they’re more likely to dismiss you because they think doing your own research makes you more of a hypochondriac.” [Participant 54]*

Additionally, provider skepticism resulted in strained patient-provider interactions; *“I can just see in the provider’s face like them reacting to these other diagnoses, they clearly think it’s ridiculous.” [Participant 42];]*; and, added complexity to finding care: *“I feel like I’ve spent a really, REALLY long time finding doctors that just believe me.” [Participant 94]* Perceptions of how healthcare providers thought about people with autonomic dysfunction along with repeated dismissal of symptoms were associated with feelings of nervousness and humiliation among interviewees: *“...to be humiliated over and over and over again- Oh, there’s nothing wrong with you.” [Participant 73]*

Others had their symptoms misattributed to psychiatric problems which resulted in feeling frustrated and dismissed. This resulted in not only dismissed: *“I just went crying from one doctor to another to help me- my fear was interpreted as an anxiety disorder…and the provider treated me like they were ‘talking to a crazy person.’” [Participant 73],* but also prolonged getting appropriate treatment. One participant summarized: *“I went undiagnosed for over a year because the doctors assumed the weight loss was due to an eating disorder not an autoimmune one. That made the diagnosis take longer than it should have. And I think people often [attribute] my stomach pain to like period cramps or something instead of being like, oh, maybe she is flaring.” [Participant 44]*

Still other participants described having their symptoms acknowledged at face value but then subsequently diminished as an expected part of life. Participants highlighted the lack of treatment or solutions that adequately addressed their issues: *“I try and seek help…It’s kind of expected for people that come back from Afghanistan like this… So it’s ‘here’s some coping exercises’, and [then] they cleared me for another deployment and I’m like, hey, I have problems.” [Participant 114].* Complex symptoms were dismissed in relation to a ubiquitous experience: *“they haven’t listened about the pain or they say ‘you know, everybody has pain.’” [Participant 94]*. Another participant reported a *“fairly dismissive”* interaction with the provider responding, *“well, you know, you’re 40”* and attributing complex symptoms to *“perimenopause and all the crazy symptoms that can be associated with perimenopause.” [Participant 42]*

Healthcare providers were sometimes described as giving physical cues, such as shrugging their shoulders in response to participant concerns – *“a shrugging of the shoulders like, oh yeah, I mean, it could be, but I don’t really know much of it,” [Participant 28]* and another participant who relayed asking a provider for their input and their response was accentuated by “*shrugging. There’s all that like shrugging.” [Participant 42]* Other dismissive healthcare providers experiences included lack of a physical exam: *“I cannot tell you how many doctors I went to that never actually physically examined me”[Participant 73]*. Participants described being passed along to another healthcare provider *“every time I go to the doctor they ping me somewhere else” [Participant 42]*.Participants completed written mental health screenings where concerning results were ignored, *“the [provider] just kind of ignores it [making it feel] more of administrative thing on their part rather than something that they actually look at.”[Participant 170]* Further, participants even described feeling that their symptoms were met with “*no empathy at all [and that they were] just completely outright ignored,” [Participant 263]* or even aggression as one participant shared that their provider’s “*response was really jarring and really scary [they] were angry that I brought [my symptoms] up.” [Participant 94]*

#### Theme 5: Positive provider experiences.

Participants also described positive experiences with healthcare providers. In contrast to having symptoms dismissed, participants felt supported when healthcare providers believed them, even in instances where they may not have definitive answers, emphasizing “*I believe you. I don’t know how I can help you. But I believe you”* and acknowledged their experiences: *“[the provider] never called me a hypochondriac or anything like that.” [Participant 582]* Time was a common factor in positive provider experiences including actual time spent with and listening to participants such as longer intentional appointments. The willingness to be “*more involved and spend more time with [the patient] and understand past and present”;* and having a provider spend time and express genuine interest *“was incredibly refreshing.”[Participant 211]* Repeatedly participants shared how impactful it was for providers to be “*willing to spend as much time with you as they need to [and be] willing to answer your questions and go over things with you.”[Participant 336]* Along with time, providers who expressed a sincere interest in learning about the patient as an individual or as one participant put it, *“treating me like a human being.”[Participant 73]* Another participant described their positive experience: *“Honestly with all the doctors at [health system], I felt like I was always listened to.”* They also highlighted, *“that’s the biggest thing for me [being] on the same path of wanting a solution, or, if not a solution, something to help me feel better.” [Participant 393]*

#### Theme 6: Experiences influenced by identity.

Participants attributed their variable experiences with healthcare providers to aspects of their personal identities, highlighting examples such as being female, young, transgender, gay, or overweight. A young female participant described the experience seeking healthcare as one in which she does not *“get taken seriously. If my leg was broken and you could see that it’s broken, I would get help for that pretty quickly, but when I go in [because I’m having] a really hard time with my mental health, it is brushed off and that’s really hard. And I feel like a part of that is like being a younger-looking cis female. Just not being taken seriously. [They say to me] ‘Oh, you’ll get over it’.” [Participant 95]* Being transgender or gay was also described as potentially contributing to having a different experience, such as the participant who described their difficult experience with providers: *“I’ve been in and out of doctors a lot, and a lot of my symptoms aren’t really physical or don’t come up on blood work. I’ve just had a lot of difficult experiences with doctors- them not believing me many times. And I’m trans, so it’s a little different of an experience and being gay at a doctor is also different.” [Participant 94]* Further, being overweight was described as a way to dismiss health problems by attributing everything to obesity: *“Medicine in general has a stigma against plus size people. And we don’t always get that adequate kind of care that you’re supposed to get because they all stem it back to ‘well you’re obese’ instead of looking at the root cause of something.” [Participant 124]*

Participants also discussed aspects of providers’ identity that seemed to relate to their care, such as gender, racial identity, and perceived cultural competency. One participant’s comments demonstrated the importance of a provider’s gender, stating: *“male doctors tend to be more dismissive of a lot of my chronic pain issues… It kind of feels like [providers who are women are] more likely to listen to my kind of struggles rather than outright dismissing them.” [Participant 54]* Another participant highlighted the importance of cultural congruence (i.e., patient and provider with a similar race/ethnicity): *“It would have been more helpful if I was with a provider who was aware, or maybe even shared like lived experiences or could relate in that way… I requested I would like to see a provider who’s a person of color at least, but I haven’t.” [Participant 122]*

#### Theme 7: Self-advocacy.

Participants described many stories of seeking care and having to “*be firm*” and “*being a detective on my own, never giving up*” *[Participant 73]* in advocating for oneself, in the face of providers dismissing them. Participants described a type of evolution in themselves, whereby at first, they just accepted they wouldn’t receive appropriate care, but then eventually the participant became their own self-advocate and demanded to be heard. However, this pushing back was described as being “*constantly frustrating [when] I try really hard to be an advocate for myself - and it just is really hard.” [Participant 44]* Participants told stories of needing to “*push for that diagnosis… I wanted to know what was wrong so then we could help treat it*.” *[Participant 114]* When asked about what advice they would give to others going through this journey, participants had ideas such as: *“The biggest thing that you can do is keep fighting to get diagnosed or to get an answer.” [Participant 350]* and *“Taking charge of your own health care and not waiting on someone else to tell you ‘okay you can go do this.’” [Participant 114]*

### Healthcare system

#### Theme 8: Systemic barriers to healthcare.

At the outermost layer of our findings is the healthcare system and most specifically, barriers to healthcare at the system level. While 30% of participants had been seen in a clinic that specialized in their most concerning symptom; 44% had never attempted to schedule an appointment at a specialty clinic. The remaining 26% were not seen because they didn’t know how to get an appointment, financial or insurance reasons, or they were determined ineligible or put on a wait list by the clinic.

Participants elaborated on long wait times to see mental health providers or other specialists. One participant described trying to seek mental health support during their cancer diagnosis, but *“we couldn’t find anybody that could fit me in for months and months and months and I kind of gave up. It is really a [problem] that when you are diagnosed with any sort of cancer, you are not assigned a psychiatrist or a therapist on your team.” [Participant 549]* Another participant described trying to see a provider recommended to them, but that *“of course, the great person isn’t available for like 6 months.” [Participant 42]* Others identified that even scheduled appointments were ‘lost in the system,’ especially if they had a status change. For example, a participant who had to switch to Medicaid was on *“waiting lists, you know, so 9 months here, 6 months here… unfortunately, at the moment I’ve been having a hard time seeing psych because… my appointments keep getting lost in the system which is extremely hard.” [Participant 341]*

Participants described feeling frustrated with the healthcare system in which they were often bounced around to different providers, each one needing to hear their story again. *“It feels like almost every appointment is a new provider so we start back at square zero. And they don’t know my history and then, even though they provide good care, we’re not moving forward…and we waste a lot of precious time…. so there’s no continuity.” [Participant 350]* This situation seems to be exacerbated with turnover of healthcare providers and having to repeatedly find new providers. Many described having a hard time starting over with different healthcare providers as they had to rebuild relationships, or felt they had to ‘start over’: *“And so that [providers leaving] makes things difficult. I start a relationship with somebody - I trust that person and then they’re gone. And so it’s difficult.” [Participant 341]* Changes in insurance coverage also forced participants to have to change healthcare providers. The anticipation of having to start over with a new provider kept participants from seeking different providers even when they were dissatisfied with their care. *“I’m on public healthcare, so I kind of just go with whoever will accept that. I don’t like my hematologist, but I see him because he takes my health care and it’s too much work to find a new one.” [Participant 44]*

Insurance coverage also impacted the care that participants were able to receive. Medicaid was mentioned as a barrier in both finding care and getting procedures approved; one participant stated that *“with me being on Medicaid, it’s kinda hard for me to find a primary care that will accept my insurance*.” *[Participant 336]* Significant delays in treatment resulted from insurance discrepancies related to obtaining prescribed medications: *“You’ll tell your doctor, ‘I need a refill’, and then they’ll put it in. And then for some reason, insurance is like, ‘this is wrong’, even though it’s the same thing… It’s exhausting.” [Participant 359]*

Participants recognized the impact of these systemic problems in healthcare and acknowledged that healthcare providers also faced systemic challenges and structural constraints. Some participants reflected that the fear that can come with conditions marked by autonomic dysfunction can create unrealistic expectations of healthcare providers who are trying to practice in a problematic healthcare system: *“I think we are so frightened as patients that we do ask too much of our doctor sometimes. The system’s not there to support doctors.” [Participant 73]* For other patients, the experiences seeking care for these conditions has resulted in a loss of faith in medicine and the healthcare system: *“I have lost a lot of faith in medicine and health care and I also I think the healthcare system is just absurd.” [Participant 42]*

### Integration of quantitative and qualitative findings

**Fig 2:**
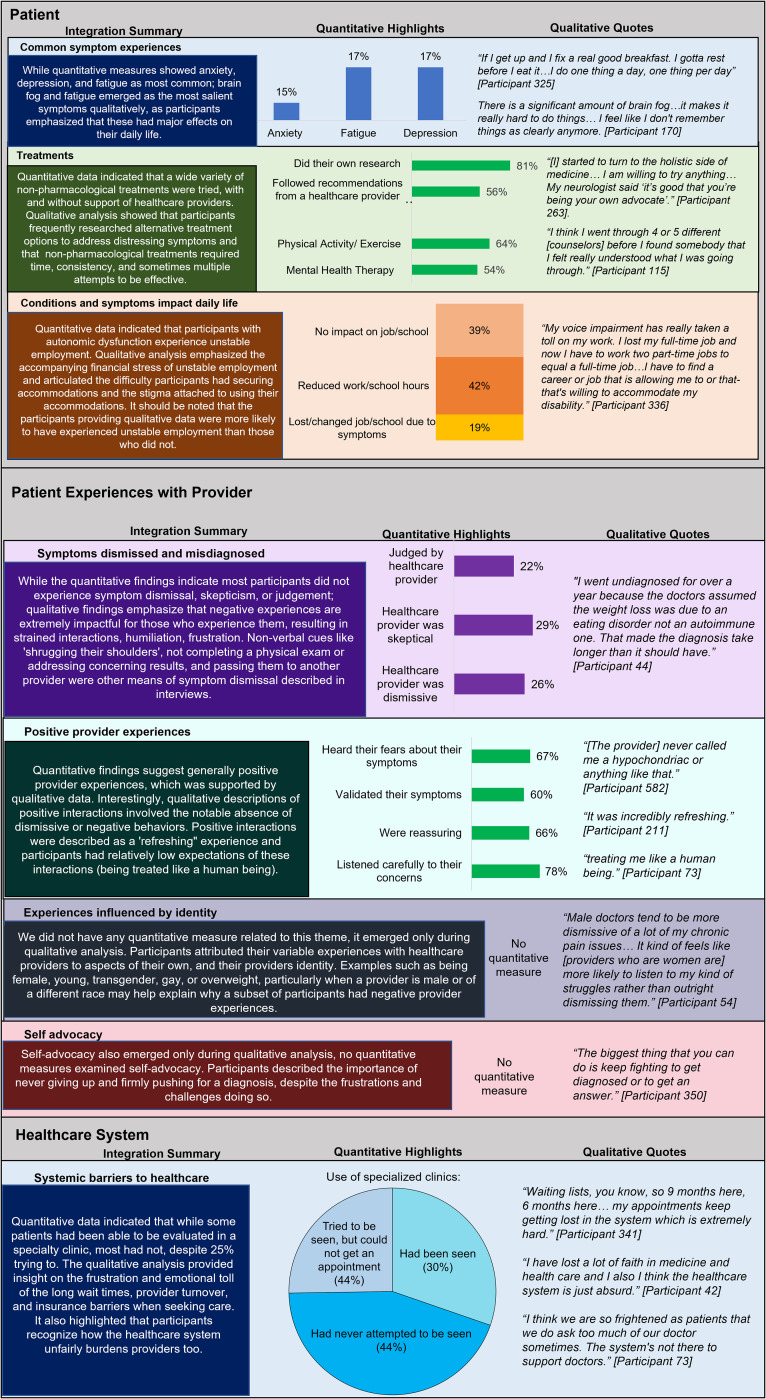
Joint display of mixed-method data. Provides a joint display of the integration of the quantitative data and qualitative data. This figure illustrates the convergence and divergence of patient experiences across three levels of the patient experience (Patient, Patient Experiences with Providers, and Healthcare System) through quantitative highlights side by side with representative qualitative quotes. Data prevalence was contrasted with qualitative salience in the integration summary. The integration summary is provided to synthesis the relationships among the data; Qualitative data provided context or details about the lived experience of autonomic dysfunction described by the quantitative results.

Quantitative data and qualitative data are integrated with a joint display in Fig 2. Overall, the quantitative results described the prevalence of various aspects of the lived experience, while the qualitative themes provided context and details about participants lived experiences with autonomic dysfunction. Two qualitative themes (Experiences influenced by identity and Self-advocacy) did not have any corresponding quantitative data, emerging solely from qualitative analysis. The joint display also highlighted the discrepancy around participants’ experiences with healthcare providers. The quantitative data suggest generally positive provider experiences (indicated by frequencies), while qualitative data emphasized more negative experiences. Deeper analysis revealed that the qualitative descriptions of positive interactions with healthcare providers relied on the notable absence of dismissive or negative behaviors: *“[the provider] never called me a hypochondriac or anything like that*.” *[Participant 582]* Positive interactions also seemed to be an almost surprising experience *“[The positive provider experience] was incredibly refreshing” [Participant 211]* as participants had relatively low expectations for these interactions *“[Positive provider experience involved] “treating me like a human being.” [Participant 73]*

## Discussion

The purpose of this study was to explore participants’ perspectives and experiences with seeking and receiving care for health symptoms consistent with autonomic dysfunction, through a mixed-methods approach with a diverse population. The limited differences between the group providing qualitative data and the group providing only quantitative data, supports our mixed-methods approach. Likewise, the intermixing of quantitative survey data and qualitative interview data resulted in findings that are highly consistent with the socioecological model public health framework [29,30], in which patients are impacted by experiences at the individual-level, at the level of interacting with a healthcare provider, and within the larger context of a healthcare system. The socioecological model was integral to our interpretation of our findings and organizing into a cohesive framework. As depicted in Figs 1 and 2, participants described experiencing common symptoms, namely fatigue, brain fog, anxiety, sleep problems, and depression, which significantly impacted several aspects of their lives, namely employment. Participants often sought non-pharmacological treatments, such as mental health support and engaging in wellness activities, and trying to be their own self-advocate for care. Participants reported turning to healthcare providers for diagnosis and management of their condition and symptoms and while many people reported having good experiences, a significant proportion of people described negative experiences with healthcare providers and the larger healthcare system. Participants in the qualitative interviews reported often feeling dismissed by providers and facing barriers at the larger systematic levels. As discovered through our mixed method approach, some experiences with healthcare providers may have positive by default, simply by not being negative. The low expectations of interactions with providers, and even surprise when interactions went well, are concerning as trust is a basic requirement of patient-provider interactions. This qualitative sample, a majority of whom identified in some way with a marginalized demographic (i.e., non-White, low-income, Medicaid/Medicare insurance coverage), identified that aspects of their personal identities, as well as the identities of their healthcare providers, may have also contributed to these negative experiences.

The impact of autonomic dysfunction on participants’ personal and professional lives was clear. For example, the impact upon employment among our participants was remarkable, with the majority reporting either a reduction in their work or school hours, having to change jobs, or losing their job or leaving school due to their condition or symptoms. Insecure employment due to health concerns was closely tied to financial stressors of increased healthcare costs in the setting of real or potential income and insurance loss. Even having or needing accommodations at work was stressful for participants, further stigmatizing many who already faced judgment due to their other characteristics.

The impacts of autonomic dysfunction were also embedded in their personal experiences with healthcare providers. While a majority of participants reported good experiences with healthcare providers, negative experiences were pervasive in interviews including “not being heard” or having their “symptoms dismissed” by healthcare providers. This widely supported qualitative theme in our interviews aligns with the concept of “symptom invalidation” by Bontempo et al (2025) [31]. For people experiencing autonomic dysfunction, being dismissed or disbelieved can undermine their own sense of bodily trust and safety, contributing to self-doubt and reduced self-advocacy [32]. Individuals from historically medically underserved populations may be particularly susceptible to experiences and consequences of not being heard. For example, a qualitative study of young women and nonbinary people with chronic illnesses found that all participants experienced “medical invalidation”, with consequences including internalization of invalidation, symptom intensification, and avoiding care [33]. Another qualitative study of 60 African American adults found frequent experiences of poor communication with healthcare providers and perceived discrimination, which contributed to medical mistrust [34]. These psychosocial impacts likely contribute to the perpetuation of autonomic dysfunction and may underlie racial and other health disparities that have been described for many conditions.

There is much literature about the concept of medically unexplained symptoms, which often coincides with autonomic dysfunction. Many of our study participants experienced the delayed diagnostic odyssey of living with medically unexplained symptoms. O’Dell et al (2025) indicate that increased recognition of the barriers that delay diagnosis is the first step in improving patient outcomes [35]. When healthcare providers do not recognize the symptoms and there are no corresponding organic findings to support a diagnosis they may conclude that the problem is not within their scope of practice, potentially dismissing the patient believing they do not have any guidance to offer. Fuss et al. (2024) acknowledge the increasing demands on healthcare providers’ day-to-day work that increase providers’ vulnerability to engaging in invalidating communication [36].

Importantly, acknowledging the harms of symptom dismissal should not be interpreted as minimizing the importance of psychiatric comorbidities. Depression, anxiety, post-traumatic stress, and other mental health conditions commonly co-occur with chronic medical illnesses and conditions marked by persistent autonomic dysfunction, often contributing meaningfully to symptom burden, quality of life, and functional impairment [37]. The challenge identified by participants in this study was not the recognition of psychiatric symptoms themselves, but rather the premature attribution of complex physical symptoms exclusively to psychiatric causes without adequate evaluation or validation of their lived experiences. These findings support integrated, interdisciplinary models of care that address both physical and psychological contributors to health while avoiding false dichotomies between “medical” and “psychiatric” explanations. Participants described genuinely positive experiences when their providers listened carefully with sincere interest, affirmed their experiences, used accessible language, spent adequate time, and provided tangible solutions and support. These findings relate to suggestions for healthcare professionals who may be diagnosing and/or treating patients with autonomic dysfunction, consistent with the extant literature. Bontempo et al. (2025) stresses that healthcare providers can experience diagnostic uncertainty yet also validate patients’ symptoms; these phenomena are not mutually exclusive [31]. While many topics that emerged from the interviews have been discussed in previous studies [31,38], the message from our participants to healthcare providers is clear: listen, validate the patient experience, recognize that normal biomedical findings do not mean the absence of symptoms, and avoid prematurely attributing symptoms solely to psychiatric or hormonal causes while remaining attentive to legitimate psychiatric comorbidities that may benefit from treatment. Ultimately, the psychological and behavioral tolls of not being heard may reinforce the physiological dysregulation at the core of many conditions, potentially creating a feedback loop of distress, invalidation, mistrust, avoidance, and increased vulnerability [21–23].

Participants in the study clearly felt most supported when the practitioner listens to them, is empathetic, and is willing to be vulnerable through expressing their own limitations. This may also correspond to ‘epistemic injustice’, a term first used in the context of the patient-practitioner relationship by Fricker in 2007 to describe practitioners not believing what patients say because of the practitioner’s biases [39]. This is particularly common when patients with chronic disease also have mental health issues. Fricker (2007) and others also recognize that patients are not given adequate credit for their claims, which results in a credibility deficit that harms them in their capacity as rational agents and as knowledgeable about their own experience [39]. This is particularly common in marginalized groups, when the way the patients describe their symptoms does not use terminology commonly utilized in medicine. The findings in this study about “having symptoms dismissed” is consistent with the literature, whereby perceived medical dismissal is associated with increased psychological distress and reduced self-esteem, particularly among individuals with chronic conditions that lack clear biological etiology [39,40], which may further exacerbate symptoms and/or impact wellbeing or functioning. A large-scale review of qualitative studies involving over 11,000 patients with ambiguous and hard-to-diagnose conditions (e.g., Long-COVID, irritable bowel syndrome, fibromyalgia, endometriosis, etc.) found that medical invalidation was associated with emotional consequences (e.g., shame, depression), healthcare-related distress (e.g., healthcare-related anxiety and trauma), avoidance of care, and diagnostic delays, which can last years and worsen outcomes [31].

Participants’ recommendations for improving interactions with healthcare providers focused primarily on creating positive interpersonal interactions rooted in dignity and respect. As one participant stated, *“People need to be treated not only for whatever ails them, but as a human being. And, they need to be looked in the eye.” [Participant 73].* Smyth and Blitshteyn (2025) detail best practices for communicating with patients with complex chronic illnesses using compassionate, encouraging, and non-judgmental language [37]. From the results of our research, we propose that healthcare providers consider these simple phrases: “I am listening,” “I believe you,” and “I don’t know, but we will work together to find answers/help.” While these actions by individual health care providers are unlikely to address systemic barriers, they can be a meaningful step to alleviating the invisibility and dismissal often experienced by patients with symptoms consistent with autonomic dysfunction.

**Fig 3:**
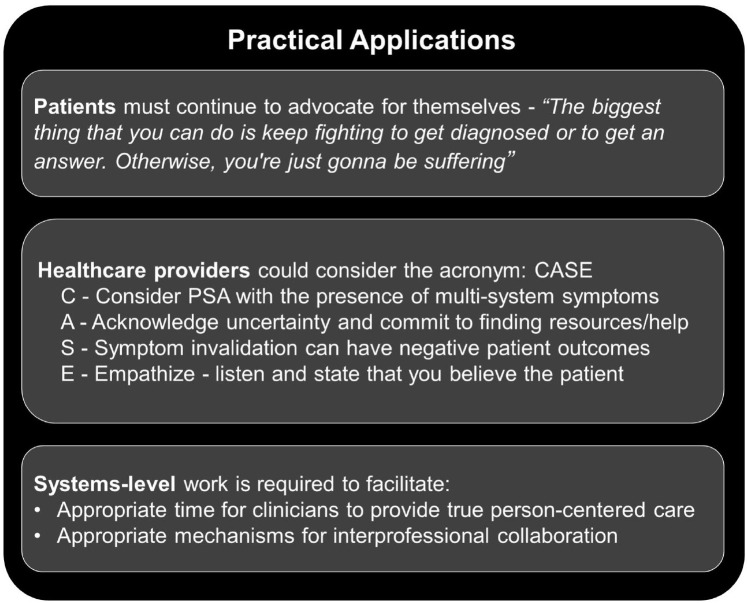
Practical applications. Presents potential practical applications of this analysis and discussion for patients, providers, and healthcare systems.

Participants in this study acknowledged that the healthcare system often creates barriers to quality care for patients *and* providers, and their recommendations above reflect this understanding. The literature has characterized the many barriers faced by healthcare practitioners [41]. Although practitioners often desire to provide a shared decision-making approach with patients, this approach may take longer than the time allotted for the visit in a high productivity and income focused healthcare system [42]. Clinicians often find themselves practicing within systems that do not allow them to practice their ideal patient-centered care, which is one cause of burnout in clinicians – a modern crisis in healthcare [43,44]. Further, patients may be anxious or have become so aversive to a healthcare system that does not meet their needs over time that they may no longer be open to the medical model. Clearly, chronic illnesses are optimally managed through an interdisciplinary biobehavioral approach [45]. However, unless this approach is framed in a way that the patient perceives as non-dismissive, patients may reject a key part of their proposed treatment, such as referral to a behavioral specialist, for fear that they are being told their symptoms are “all in their head.” [46] Still, patients fare best when they take ownership of their health. The theme of self-advocacy identified in this study, complements the critical need for patients to actively engage in and manage their own symptoms healthcare described in the literature [13,47]. Fig 3 provides suggested practical applications for addressing the challenges identified in this study for patients, healthcare providers, and health systems.

When faced with chronic illness, participants in this study described a continuum of challenges with accessibility to the healthcare system. These challenges ranged from no access to healthcare at all, access to healthcare being perceived as untrustworthy because practitioners do not appear to believe symptoms are “real”, non-equitable treatment for certain groups or because of perceived limited levels of competence, and to access to trusted, responsive and high-quality care. Our data support a complex construct of the definition and meaning of “access to healthcare” for people with conditions reflecting autonomic dysfunction [48]. Some have no physical access due to resource limitations, some have no meaningful access due to practitioner beliefs and attitudes, and others may disconnect themselves from the healthcare system due to negative experiences. Each of these requires a different approach to help provide true beneficial access to all healthcare recipients and close attention to these is warranted in the research and clinical arenas in this area.

### Future research

Healthcare providers and healthcare systems would benefit from understanding the outcomes associated with these phenomena and interventions that address the underlying biological mechanisms of autonomic dysfunction. While our study explored a heterogenous population linked primarily through shared symptom experiences, sub-group analysis (based on diagnoses or demographic characteristics) may provide further insight into these experiences and support more personalized interventions. Given the established consequences of symptom and medical invalidation, future work may explore interventions, whether clinical, relational, or systemic, that aim to restore self-trust and/or healthcare relationships following these experiences. Future research should also further examine the mechanisms through which perceived medical invalidation contributes to psychological distress and physiological dysregulation, particularly in conditions marked by autonomic dysfunction. Additionally, incorporating well established biomarkers of autonomic function (e.g., heart rate variability [HRV]) in research on medical invalidation could generate further insight into the psychophysiological impact of these encounters and guide more holistic care models.

An example of an established tangible solution that providers should consider presenting to patients is the use of remote measurement technologies such as commercial wearable devices that measure HRV [49]. HRV provides a non-invasive, validated digital biomarker of autonomic activity and the opportunity to visualize these otherwise invisible symptoms and conditions marked by autonomic dysfunction [50–52] These interventions have the potential to transform the care of people with conditions marked by autonomic dysfunction, which is a key area to target for future research.

Future work focused on communication, such as Patient-Centered Care, could serve as the foundation for how healthcare providers care for this population; this approach improves patient outcomes [31], but our participants’ accounts tell us that this is not yet the standard of care. There is limited research on healthcare providers’ perspectives on caring for patients with symptoms associated with autonomic dysfunction, and future studies should investigate the clinical resources, knowledge, and support that healthcare providers need to improve outcomes for those with symptoms of autonomic dysfunction. Finally, an opportunity for future work emerged during qualitative analysis as patients described the personal evolution they underwent throughout their healthcare seeking journeys. Further understanding of the dynamic qualities of patients’ lived experiences in relation to their health and healthcare remains a valuable aim for future research.

### Limitations

Given our local recruitment efforts, participants in this study were primarily from central Virginia, which is home to several health systems and a diverse population. While the mixed-method approach provided contextual insight to our findings, the voluntary nature of our sampling strategy may have introduced bias toward individuals with more extreme experiences. Furthermore, the qualitative data was provided by a group of people who wanted to share their stories, which may also reflect those who had more extreme experiences at the personal, provider, and system levels that should not be generalized to all people experiencing autonomic dysfunction. Additionally, it is unknown if the experiences of people in our sample would differ from the experiences of those from different regions or in different health systems (particularly those outside the United States) or those from other populations. All data was self-reported and we did not compare against any secondary source such as the electronic health record. Specifically, an important limitation is that autonomic dysfunction was not directly measured using physiological or autonomic biomarkers. Participants were included based on self-reported symptoms and diagnoses that have been associated with autonomic dysfunction in prior literature, rather than standardized autonomic testing. As a result, we cannot conclude that all participants were experiencing objectively verified autonomic dysregulation at the time of participation, nor can we determine the degree to which autonomic dysfunction contributed to individual symptoms. The purpose of this study was to understand the lived experiences of individuals who identify with conditions and symptom presentations commonly associated with autonomic dysfunction; however, future research would benefit from incorporating objective measures of autonomic function, such as heart rate variability, autonomic reflex testing, tilt table testing, or other validated physiological markers, to better characterize the relationship between subjective experiences and autonomic dysregulation.

Conceptually, we recognize that autonomic dysfunction and related concepts including persistent sympathetic activation (PSA), dysautonomia, chronic stress, and allostatic load are still being disentangled and defined in the literature and in practice as is our understanding of the pathophysiology of conditions associated with autonomic dysfunction. The evolving conversation [53,54] and understanding of these constructs may limit the generalization of our findings to all individuals living with the reported conditions and symptoms of the participants in this study, however we believe that the lived experience of our participants may translate to others regardless of evolving definitions and understanding of physiological pathways. Of note in light of limitations, however, our robust sample size, recruited from a geographic region that covers rural and urban communities, and standardized methodology help to mitigate the impact of these limitations.

## Conclusions

The lived experience for people with autonomic dysfunction is multilayered and is associated with the patient-provider relationship embedded in unsupportive healthcare systems. These experiences may reinforce the physiological dysregulation at the core of autonomic dysfunction, potentially creating a feedback loop of distress, invalidation, mistrust, avoidance, and increased vulnerability.

## Supporting information

S1 Table Consolidated criteria for reporting qualitative studies (COREQ): 32-item checklist. The qualitative portion of this mixed-method study meets reporting requirements in all three domains of the COREQ [28]. The reported page number for each item corresponds to where each item can be located within the manuscript.
(PDF)
