# Supplementary material for: Lived experiences and perspectives of persons with conditions marked by autonomic dysfunction
Source: PLoS One. 2026 Aug 17;21(8):e0355952. doi: 10.1371/journal.pone.0355952 (PMC13480646; doi:10.1371/journal.pone.0355952)
Supplement: Supplementary file 2 [file pone.0355952.s003.docx]

July 6, 2026

Dr. Grubić Kezele

Academic Editor

PLOS One

Dear Dr. Grubić Kezele,

We thank you and the insightful review of our article (PONE-D-25-59300) entitled *“Lived Experiences and Perspectives of Persons with Conditions Marked by Persistent Sympathetic Activation”*. Our revisions are noted as track-changes within the revised manuscript and are summarized below (Table 1). A clean copy of our revised manuscript is also included in our submission.

Thank you for your suggestions and insights to ensure our manuscript meets journal requirements, addresses statistical analyses concerns, and strengthens the scientific rigor, transparency, and interpretability of our findings. We are hopeful that our revisions have offered conceptual clarification and strengthened the methodological transparency of our manuscript.

Sincerely,

Lisa L. Shah PhD, RN

Assistant Professor

VCU School of Nursing

Table 1. Responses to Editorial and Reviewer Comments

| **Reviewer Comment** | **Response to Reviewer** |
| --- | --- |
| ***Journal Requirements*** | |
| 1. Please ensure that your manuscript meets PLOS ONE's style requirements, including those for file naming | We reviewed our manuscript to ensure adherence to the journal’s style requirements. |
| 2. In the ethics statement in the Methods, you have specified that verbal consent was obtained. Please provide additional details regarding how this consent was documented and witnessed, and state whether this was approved by the IRB | In the Methods, participants section, we have clarified that the verbal consent was completed with the research team. IRB approval of the entire protocol is indicated in the introduction of the Methods section. |
| 3. We note that the grant information you provided in the ‘Funding Information’ and ‘Financial Disclosure’ sections do not match. When you resubmit, please ensure that you provide the correct grant numbers for the awards you received for your study in the ‘Funding Information’ section. | The conflict of interest statement has been updated to match the Funding Information section. |
| 4. We note that your Data Availability Statement is currently as follows: “All relevant data are within the manuscript and its Supporting Information files.” Please confirm at this time whether or not your submission contains all raw data required to replicate the results of your study. Authors must share the “minimal data set” for their submission. PLOS defines the minimal data set to consist of the data required to replicate all study findings reported in the article, as well as related metadata and methods (<https://journals.plos.org/plosone/s/data-availability#loc-minimal-data-set-definition>).  For example, authors should submit the following data:  - The values behind the means, standard deviations and other measures reported; - The values used to build graphs; - The points extracted from images for analysis.  Authors do not need to submit their entire data set if only a portion of the data was used in the reported study.  If your submission does not contain these data, please either upload them as Supporting Information files or deposit them to a stable, public repository and provide us with the relevant URLs, DOIs, or accession numbers. For a list of recommended repositories, please see <https://journals.plos.org/plosone/s/recommended-repositories>.  If there are ethical or legal restrictions on sharing a de-identified data set, please explain them in detail (e.g., data contain potentially sensitive information, data are owned by a third-party organization, etc.) and who has imposed them (e.g., an ethics committee). Please also provide contact information for a data access committee, ethics committee, or other institutional body to which data requests may be sent. If data are owned by a third party, please indicate how others may request data access. | We have added a Data availability statement as follows:  “Data for this study is not publicly available due to ethical restrictions. Participants in this study did not consent to having their data shared publicly, and thus the IRB protocol does not allow public sharing. However, data can be shared upon request with an approved data use agreement (DUA) and ethical approval. To request data, please contact the corresponding author at [shahL2@vcu.edu](mailto:shahL2@vcu.edu)”  In our preparation of a shareable dataset we noted that analysis in part of Table 4 had inadvertently excluded some participants. We have corrected the analysis and updated Table 4. The updated results were not significantly different from the originally shared results. |
| ***Reviewer Comments*** | |
| *1. Definition and operationalization of PSA:* The manuscript uses persistent sympathetic activation (PSA) as a unifying construct across highly heterogeneous conditions (e.g anxiety, cancer, POTS, long-COVID). The authors should more clearly justify PSA as a shared underlying mechanism rather than a descriptive umbrella term, and discuss the risks of conceptual over-generalization. | Through team discussions, we have decided that the term autonomic dysfunction, rather than persistent sympathetic activation (PSA), is the best concept to represent our sample. This decision was driven by the still evolving conversation on terminology (Crouch et al., Benarroch, 2021) and our team’s recognition of limitations of PSA as a standalone concept, without consideration of the simultaneous withdrawal of the parasympathetic response and co-occurring neuroendocrine and immune responses. We have revised our manuscript to primarily use the term autonomic dysfunction, conceptualized as an underlying mechanism of many conditions and symptoms. |
| *2. Distinction between PSA and related constructs:*  Please clarify how PSA is conceptually distinct from related constructs such as chronic stress, HPA-axis dysregulation, allostatic load, or medically unexplained symptoms. A brief conceptual framework would improve clarity. | As acknowledged in the previous comment, we have carefully considered our terminology and as a team have agreed that autonomic dysfunction is the most accurate concept for this study. In doing so, we recognize the conceptual blurring of many related constructs identified by the reviewer and in the literature. We have acknowledged the evolving conceptual understanding of these terms in our limitations section:  “Conceptually, we recognize that autonomic dysfunction and related concepts including persistent sympathetic activation (PSA), dysautonomia, chronic stress, and allostatic load are still being disentangled and defined in the literature and in practice as is our understanding of the pathophysiology of conditions associated with autonomic dysfunction. The evolving conversation and understanding of these constructs may limit the generalization of our findings to all individuals living with the reported conditions and symptoms of the participants in this study, however we believe that the lived experience of our participants may translate to others regardless of evolving definitions and understanding of physiological pathways.” |
| *3. Theoretical grounding of the socioecological framework:* While the socioecological model is referenced, its integration into the study design and analysis appears largely post-hoc. Please clarify how this framework informed data collection, coding, or interpretation a priori. | We have clarified that the SEM was used to inform interpretation of our findings:  “The socioecological model was integral to our interpretation of our findings and organizing into a cohesive framework. ” |
| *4. Heterogeneity of the study population:* The inclusion of participants with vastly different diagnoses (psychiatric, neurological, oncological, autonomic) raises concerns about internal coherence. The authors should discuss how this heterogeneity affects interpretability and whether subgroup analyses were considered. | In the Future research section, we acknowledge the heterogeneity of our population and suggest that subgroup analyses could be done in the future. We considered various potential subgroup analyses based on symptom, diagnoses, and several demographic variables, but ultimately agreed that the common experience of symptoms marked by PSA was important to understand broadly, regardless of patient or disease characteristic. We added the following to address the concern:  “While our study explored a heterogenous population linked primarily through PSA symptom experience, sub-group analysis (based on diagnoses or demographic characteristics) may provide further insight into these experiences.” |
| *5. Reliance on self-identified PSA:* PSA is inferred entirely from self-reported symptoms or diagnoses. Please more explicitly acknowledge the limitations of this approach and clarify whether any standardized autonomic criteria were considered or could be integrated in future studies. | We have revised the Limitations section to explicitly acknowledge the limitations of reliance on self-identified PSA and have clarified our rationale for using self-report without standardized autonomic criteria.  “Specifically, an important limitation is that PSA was not directly measured using physiological or autonomic biomarkers. Participants were included based on self-reported symptoms and diagnoses that have been associated with persistent sympathetic activation in prior literature, rather than standardized autonomic testing. As a result, we cannot conclude that all participants were experiencing objectively verified autonomic dysregulation at the time of participation, nor can we determine the degree to which sympathetic activation contributed to individual symptoms. The purpose of this study was to understand the lived experiences of individuals who identify with conditions and symptom presentations commonly associated with PSA; however, future research would benefit from incorporating objective measures of autonomic function, such as heart rate variability, autonomic reflex testing, tilt table testing, or other validated physiological markers, to better characterize the relationship between subjective experiences and autonomic dysregulation.” |
| *6. Recruitment via ICD codes and self-selection bias:* Recruitment through ICD codes combined with voluntary survey participation may bias the sample toward individuals with more negative healthcare experiences. This potential bias should be discussed more explicitly. | We have updated our limitations section to discuss this potential bias explicitly.  “While the mixed-method approach provided contextual insight to our findings, the voluntary nature of our sampling strategy may have introduced bias toward individuals with more extreme experiences. Furthermore, the qualitative data was provided by a group of people who wanted to share their stories, which may also reflect more extreme experiences at the personal, provider, and system levels that should not be generalized to all people experiencing PSA.” |
| *7. Marginalized populations focus:* The manuscript emphasizes marginalized populations, but the analytic strategy does not systematically compare marginalized vs. non-marginalized groups. Please clarify whether this was an exploratory or descriptive aim only. | Our focus on recruiting marginalized populations was exploratory as our intent was to learn about PSA and clarify understanding of experiences among people who are not historically included in research, but who do carry the burden of disease. We did not systematically compare marginalized vs. non-marginalized in this study as we need to first understand the broader lived experience and perspectives of people with conditions marked by PSA. Additionally, grouping our participants as marginalized vs. non-marginalized is not reliable as many of our participants felt marginalized in their clinical encounters, that may not align with demographic ‘markers’ of marginalization. We feel the exploratory nature of the study better supports looking at the sample as a whole, rather than dividing into subgroups without more data supporting such divisions. |
| *8. Justification of survey instruments:* Only the Fatigue Severity Scale is clearly referenced. Please clarify whether other survey items were adapted, validated, or investigator-developed, and provide justification for their use. | We have clarified that all survey instruments were investigator developed except for the Fatigue Severity Scale.  “The investigator developed survey gathered information about participant characteristics, symptoms, including the validated Fatigue Severity Scale [23], non-pharmacological treatments, impacts on employment or schooling, and experiences with healthcare providers and the healthcare system.” |
| *9. Handling of missing data:* Participants with missing data were excluded from specific analyses. Please report the extent of missingness and discuss whether missing data were random. | We have clarified how missing data was handled in the Data analysis section:  “Each variable was analyzed using non-missing records; the number of non-missing records for each is listed in each table.”  We also addressed the extent and randomness of missing data in the Results section:  “Minimal data was missing and determined to be random with non-significant influence on findings.” |
| 1*0. Statistical comparisons between interview and survey-only groups:* The manuscript reports limited differences between groups. Please clarify why these comparisons were conducted and how they informed the mixed-methods integration. | We compared the interview and survey-only groups to explore how well our interview sample demographically represented the larger survey sample. Given the minimal differences we feel this supports our mixed-method approach. This is clarified in the Discussion:  “ The limited differences between the group providing qualitative data and the group providing only quantitative data, supports our mixed-methods approach.” |
| *11. Sampling strategy for interviews:* Please clarify how the subset of 45 interview participants was selected from survey respondents and whether saturation was formally assessed. | The sampling strategy for interviews has been described in the Methods/ Interview section:  “Email invitations were sent to interested individuals, with targeted sampling of members of underserved populations (e.g., Medicaid, non-white) to be demographically representative of the survey sample “ |
| *12. Reflexivity and positionality:* While analyst triangulation is described, the manuscript would benefit from a brief reflexivity statement addressing researchers’ disciplinary backgrounds and potential influence on interpretation. | We have added a Reflexivity and positionality statement:  “In alignment with mixed-methods quality frameworks, we explicitly acknowledge our positionality. The primary analysts (LS, PK, SM, AR, RD, LL, LM, AS) are all nurses and researchers. Our multidisciplinary co-authors including physician scientists (RG, GC, TC), biostatistics (LK, NB), and psychology (TBC) also critically reviewed our study throughout its design, analysis, and interpretation to challenge and enhance our insights and assumptions. Our prior experience working with patients with a range of conditions and symptoms provided valuable contextual insight during the qualitative interview phase but also introduced potential assumptions regarding what type of quantitative analyses were considered. To mitigate potential bias during data integration and analysis, we utilized an audit trail, analyst triangulation, peer debriefing (EP), and frequent discussions with our multi-disciplinary team to ensure that the integrated findings accurately reflects the data.” |
| *13. Use of COREQ checklist:* The authors cite COREQ but do not explicitly indicate adherence. Please clarify whether a completed COREQ checklist is available or can be included as supplementary material. | We have completed the COREQ checklist and attached as supplementary material, noting the addition of the completed COREQ checklist in the Data Analysis section. |
| *14. Balance between participant quotes and interpretation:* Some sections rely heavily on extended quotations with limited analytic synthesis. Consider strengthening interpretive commentary to better link quotes to overarching themes. | We have shifted our balance of participant quotations and analytic synthesis throughout the results section (noted in tracked changes). The themes with the most substantial changes are:   - Common symptom experiences (after table 2 in regards to fatigue and after Table 3 in regards to non-pharmacological treatments) - Conditions and symptoms impact life - Patients experience with healthcare - Symptoms dismissed and misdiagnosed - Positive provider experiences - Self advocacy |
| *15. Mixed-methods integration strategy:* The manuscript states that qualitative and quantitative findings were integrated, but the integration appears largely narrative. Please clarify whether a formal mixed-methods integration approach (e.g., joint displays, convergence coding) was used. | We have created a joint display as Figure 3. The joint display shows the quantitative and qualitative data side by side, along with a summary of how the methods integrated. We have also included the development of this in our Methods Section |
| *16. Alignment between quantitative and qualitative findings:* Quantitative data suggest generally positive provider experiences, whereas qualitative data emphasize dismissal and mistrust. This apparent discrepancy deserves deeper analytic exploration. | The joint display, now included as Figure 3 providers deeper analytic exploration of the discrepancy between qualitative and quantitative data in terms of positive vs negative experiences. We have also included this in our methods, discussion, and as a new section (Integration of quantitative and qualitative findings) in results:  “Quantitative data and qualitative data are integrated with a joint display in Figure 2. Overall, the quantitative results described the prevalence of various aspects of the lived experience, while the qualitative themes provided context and details about participants lived experiences with autonomic dysfunction. Two qualitative themes (Experiences influenced by identity and Self-advocacy) did not have any corresponding quantitative data, emerging solely from qualitative analysis. The joint display also highlighted the discrepancy around participants' experiences with healthcare providers. The quantitative data suggest generally positive provider experiences (indicated by frequencies), while qualitative data emphasized more negative experiences. Deeper analysis revealed that the qualitative descriptions of positive interactions with healthcare providers relied on the notable absence of dismissive or negative behaviors: *“[the provider] never called me a hypochondriac or anything like that*.” [Participant 582] Positive interactions also seemed to be an almost surprising experience *"[The positive provider experience] was incredibly refreshing”[Participant 211]* as participants had relatively low expectations for these interactions *"[Positive provider experience involved] “treating me like a human being.” [Participant 73]* |
| *17. Causal language:* Some discussion sections imply causal pathways (e.g., healthcare invalidation reinforcing PSA). Given the cross-sectional design, causal language should be tempered. | We have reviewed and carefully edited our discussion and results to accurately reflect the cross-sectional nature of our study and to avoid implying causation. We do believe the statements about invalidation reinforcing PSA are accurate, and have added citations supporting this statement. |
| *18. Psychiatric comorbidity interpretati*on:  The manuscript appropriately cautions against misattributing symptoms to psychiatric causes, but should also more clearly acknowledge legitimate psychiatric comorbidities and the need for integrated care. | We have added detail to the discussion about psychiatric comorbidities for clarification:  “Importantly, acknowledging the harms of symptom dismissal should not be interpreted as minimizing the importance of psychiatric comorbidities. Depression, anxiety, post-traumatic stress, and other mental health conditions commonly co-occur with chronic medical illnesses and conditions marked by persistent sympathetic activation, often contributing meaningfully to symptom burden, quality of life, and functional impairment [35]. The challenge identified by participants in this study was not the recognition of psychiatric symptoms themselves, but rather the premature attribution of complex physical symptoms exclusively to psychiatric causes without adequate evaluation or validation of their lived experiences. These findings support integrated, interdisciplinary models of care that address both physical and psychological contributors to health while avoiding false dichotomies between “medical” and “psychiatric” explanations”  As well as in the message from our participants to healthcare providers:  “...prematurely attributing symptoms solely to psychiatric or hormonal causes while remaining attentive to legitimate psychiatric comorbidities that may benefit from treatment.” |
| *19. Use of “medical invalidation” and “epistemic injustice”:* These concepts are compelling but philosophically loaded. Please clarify how they are operationalized empirically in this study to avoid over-interpretation. | We have reviewed our use of the terms ‘medical invalidation’ and ‘epistemic injustice’ and have tempered the language to avoid over-interpretation. We did not operationalize these concepts in our study, but rather found alignment of our findings with the way these concepts are described in the literature. |
| *20. Clinical recommendations exceed evidence base:* Suggestions such as HRV monitoring via wearables are interesting but speculative. Please clearly distinguish evidence-based findings from future-oriented hypotheses. | We have updated our future research section to distinguish between evidence based, established findings and future oriented work. |
| 21. Provider burden and system constraints:  While systemic barriers are acknowledged, the discussion could better balance patient experiences with structural constraints faced by clinicians to avoid unintended provider-blaming narratives. | We edited our results (Systemic barriers to health section) and discussion to introduce a better balance of the structural constraints faced by healthcare providers to avoid provider blaming:  “Participants recognized the impact of these systemic problems in healthcare and acknowledged that healthcare providers also faced systemic challenges and structural constraints.”  “Still, patients fare best when they take ownership of their health. The theme of self-advocacy identified in this study, complements the critical need for patients to actively engage in and manage their own symptoms healthcare described in the literature”.” |
| *22. Figures clarity:* Figures 1 and 2 are conceptually helpful but would benefit from more detailed captions explaining how they were derived from the data. | Figure 1 and 2 have been revised and details captions have been added. |
| *23. Terminology consistency:* Please ensure consistent use of terms such as PSA, autonomic dysfunction, and sympathetic activation throughout the manuscript. | We have reviewed to ensure the term PSA is used consistently throughout the manuscript. |
| *24. Limitations section expansion:* The limitations section is appropriate but should further address recall bias, self-report bias, and the lack of objective physiological measures. | We have added detail to the limitations section to address potential biases and the lack of physiological measures. (See notes for points #5 and #6). |
| *25. Generalizability:* Please more clearly delineate which findings are likely context-specific (central Virginia, U.S. healthcare system) versus potentially transferable to other settings. | Our limitation section states that the extent of transferability is unknown. We don’t believe we have the ability to confidently state that any findings are context specific, as our understanding of these experiences is under-developed. We did clarify that health systems outside of the U.S. may have particularly different experiences that what we found. |
