# Supplementary material for: Lived experiences and perspectives of persons with conditions marked by autonomic dysfunction
Source: PLoS One. 2026 Aug 17;21(8):e0355952. doi: 10.1371/journal.pone.0355952 (PMC13480646; doi:10.1371/journal.pone.0355952)
Supplement: Peer review history

# Peer review: Decision Letter 0

**DOI:** 10.1371/journal.pone.0355952.r001

**Authors:** Tanja Grubić Kezele

7 May 2026

Dear Dr. Shah,

Thank you for submitting your manuscript to PLOS ONE. After careful consideration, we feel that it has merit but does not fully meet PLOS ONE’s publication criteria as it currently stands. Therefore, we invite you to submit a revised version of the manuscript that addresses the points raised during the review process.

Please submit your revised manuscript by Jun 21 2026 11:59PM. If you will need more time than this to complete your revisions, please reply to this message or contact the journal office at plosone@plos.org. When you're ready to submit your revision, log on to https://www.editorialmanager.com/pone/ and select the 'Submissions Needing Revision' folder to locate your manuscript file.

- A letter that responds to each point raised by the academic editor and reviewer(s). You should upload this letter as a separate file labeled 'Response to Reviewers'.
- A marked-up copy of your manuscript that highlights changes made to the original version. You should upload this as a separate file labeled 'Revised Manuscript with Track Changes'.
- An unmarked version of your revised paper without tracked changes. You should upload this as a separate file labeled 'Manuscript'.

If you would like to make changes to your financial disclosure, please include your updated statement in your cover letter. Guidelines for resubmitting your figure files are available below the reviewer comments at the end of this letter.

If applicable, we recommend that you deposit your laboratory protocols in protocols.io to enhance the reproducibility of your results. Protocols.io assigns your protocol its own identifier (DOI) so that it can be cited independently in the future. For instructions see: https://journals.plos.org/plosone/s/submission-guidelines#loc-laboratory-protocols. Additionally, PLOS ONE offers an option for publishing peer-reviewed Lab Protocol articles, which describe protocols hosted on protocols.io. Read more information on sharing protocols at https://plos.org/protocols?utm_medium=editorial-email&utm_source=authorletters&utm_campaign=protocols.

As the corresponding author, your ORCID iD is verified in the submission system and will appear in the published article. PLOS supports the use of ORCID, and we encourage all coauthors to register for an ORCID iD and use it as well. Please encourage your coauthors to verify their ORCID iD within the submission system before final acceptance, as unverified ORCID iDs will not appear in the published article. *Only*  the individual author can complete the verification step; PLOS staff *cannot*  verify ORCID iDs on behalf of authors.

We look forward to receiving your revised manuscript.

Kind regards,

Tanja Grubić Kezele, Ph.D., M.D.

Academic Editor

PLOS One

Journal Requirements:

When submitting your revision, we need you to address these additional requirements.

1. Please ensure that your manuscript meets PLOS ONE's style requirements, including those for file naming. The PLOS ONE style templates can be found at

https://journals.plos.org/plosone/s/file?id=wjVg/PLOSOne_formatting_sample_main_body.pdf and

https://journals.plos.org/plosone/s/file?id=ba62/PLOSOne_formatting_sample_title_authors_affiliations.pdf

2. In the ethics statement in the Methods, you have specified that verbal consent was obtained. Please provide additional details regarding how this consent was documented and witnessed, and state whether this was approved by the IRB

3. We note that the grant information you provided in the ‘Funding Information’ and ‘Financial Disclosure’ sections do not match.

When you resubmit, please ensure that you provide the correct grant numbers for the awards you received for your study in the ‘Funding Information’ section.

4. We note that your Data Availability Statement is currently as follows:

“All relevant data are within the manuscript and its Supporting Information files.”

Please confirm at this time whether or not your submission contains all raw data required to replicate the results of your study. Authors must share the “minimal data set” for their submission. PLOS defines the minimal data set to consist of the data required to replicate all study findings reported in the article, as well as related metadata and methods (https://journals.plos.org/plosone/s/data-availability#loc-minimal-data-set-definition).

For example, authors should submit the following data:

- The values behind the means, standard deviations and other measures reported;

- The values used to build graphs;

- The points extracted from images for analysis.

Authors do not need to submit their entire data set if only a portion of the data was used in the reported study.

If your submission does not contain these data, please either upload them as Supporting Information files or deposit them to a stable, public repository and provide us with the relevant URLs, DOIs, or accession numbers. For a list of recommended repositories, please see https://journals.plos.org/plosone/s/recommended-repositories.

If there are ethical or legal restrictions on sharing a de-identified data set, please explain them in detail (e.g., data contain potentially sensitive information, data are owned by a third-party organization, etc.) and who has imposed them (e.g., an ethics committee). Please also provide contact information for a data access committee, ethics committee, or other institutional body to which data requests may be sent. If data are owned by a third party, please indicate how others may request data access.

5. If the reviewer comments include a recommendation to cite specific previously published works, please review and evaluate these publications to determine whether they are relevant and should be cited. There is no requirement to cite these works unless the editor has indicated otherwise.

**Additional Editor Comments:**

Based on the reviewers' suggestions, the paper needs major revision. The reviewers' comments can be found below.

[Note: HTML markup is below. Please do not edit.]

Reviewers' comments:

Reviewer's Responses to Questions

**Comments to the Author**

1. Is the manuscript technically sound, and do the data support the conclusions?

Reviewer #1: Yes

**********

2. Has the statistical analysis been performed appropriately and rigorously?

Reviewer #1: No

**********

3. Have the authors made all data underlying the findings in their manuscript fully available?

The PLOS Data policy

Reviewer #1: Yes

**********

4. Is the manuscript presented in an intelligible fashion and written in standard English?

Reviewer #1: Yes

**********

Reviewer #1: Dear Editor

Thank you for the opportunity the review this paper. The manuscript addresses an important and underexplored topic the lived experiences of individuals with conditions marked by persistent sympathetic activation (PSA) using a mixed-methods design with a strong focus on marginalized populations. The study is timely, socially relevant, and largely well executed. However, several conceptual, methodological, and reporting issues should be addressed to strengthen scientific rigor, transparency, and interpretability.

Major Comments

1. Definition and operationalization of PSA

The manuscript uses persistent sympathetic activation (PSA) as a unifying construct across highly heterogeneous conditions (e.g anxiety, cancer, POTS, long-COVID). The authors should more clearly justify PSA as a shared underlying mechanism rather than a descriptive umbrella term, and discuss the risks of conceptual over-generalization.

2. Distinction between PSA and related constructs

Please clarify how PSA is conceptually distinct from related constructs such as chronic stress, HPA-axis dysregulation, allostatic load, or medically unexplained symptoms. A brief conceptual framework would improve clarity.

3. Theoretical grounding of the socioecological framework

While the socioecological model is referenced, its integration into the study design and analysis appears largely post-hoc. Please clarify how this framework informed data collection, coding, or interpretation a priori.

4. Heterogeneity of the study population

The inclusion of participants with vastly different diagnoses (psychiatric, neurological, oncological, autonomic) raises concerns about internal coherence. The authors should discuss how this heterogeneity affects interpretability and whether subgroup analyses were considered.

5. Reliance on self-identified PSA

PSA is inferred entirely from self-reported symptoms or diagnoses. Please more explicitly acknowledge the limitations of this approach and clarify whether any standardized autonomic criteria were considered or could be integrated in future studies.

6. Recruitment via ICD codes and self-selection bias

Recruitment through ICD codes combined with voluntary survey participation may bias the sample toward individuals with more negative healthcare experiences. This potential bias should be discussed more explicitly.

7. Marginalized populations focus

The manuscript emphasizes marginalized populations, but the analytic strategy does not systematically compare marginalized vs. non-marginalized groups. Please clarify whether this was an exploratory or descriptive aim only.

8. Justification of survey instruments

Only the Fatigue Severity Scale is clearly referenced. Please clarify whether other survey items were adapted, validated, or investigator-developed, and provide justification for their use.

9. Handling of missing data

Participants with missing data were excluded from specific analyses. Please report the extent of missingness and discuss whether missing data were random.

10. Statistical comparisons between interview and survey-only groups

The manuscript reports limited differences between groups. Please clarify why these comparisons were conducted and how they informed the mixed-methods integration.

11. Sampling strategy for interviews

Please clarify how the subset of 45 interview participants was selected from survey respondents and whether saturation was formally assessed.

12. Reflexivity and positionality

While analyst triangulation is described, the manuscript would benefit from a brief reflexivity statement addressing researchers’ disciplinary backgrounds and potential influence on interpretation.

13. Use of COREQ checklist

The authors cite COREQ but do not explicitly indicate adherence. Please clarify whether a completed COREQ checklist is available or can be included as supplementary material.

14. Balance between participant quotes and interpretation

Some sections rely heavily on extended quotations with limited analytic synthesis. Consider strengthening interpretive commentary to better link quotes to overarching themes.

15. Mixed-methods integration strategy

The manuscript states that qualitative and quantitative findings were integrated, but the integration appears largely narrative. Please clarify whether a formal mixed-methods integration approach (e.g., joint displays, convergence coding) was used.

16. Alignment between quantitative and qualitative findings

Quantitative data suggest generally positive provider experiences, whereas qualitative data emphasize dismissal and mistrust. This apparent discrepancy deserves deeper analytic exploration.

17. Causal language

Some discussion sections imply causal pathways (e.g., healthcare invalidation reinforcing PSA). Given the cross-sectional design, causal language should be tempered.

18. Psychiatric comorbidity interpretation

The manuscript appropriately cautions against misattributing symptoms to psychiatric causes, but should also more clearly acknowledge legitimate psychiatric comorbidities and the need for integrated care.

19. Use of “medical invalidation” and “epistemic injustice”

These concepts are compelling but philosophically loaded. Please clarify how they are operationalized empirically in this study to avoid over-interpretation.

20. Clinical recommendations exceed evidence base

Suggestions such as HRV monitoring via wearables are interesting but speculative. Please clearly distinguish evidence-based findings from future-oriented hypotheses.

21. Provider burden and system constraints

While systemic barriers are acknowledged, the discussion could better balance patient experiences with structural constraints faced by clinicians to avoid unintended provider-blaming narratives.

22. Figures clarity

Figures 1 and 2 are conceptually helpful but would benefit from more detailed captions explaining how they were derived from the data.

23. Terminology consistency

Please ensure consistent use of terms such as PSA, autonomic dysfunction, and sympathetic activation throughout the manuscript.

24. Limitations section expansion

The limitations section is appropriate but should further address recall bias, self-report bias, and the lack of objective physiological measures.

25. Generalizability

Please more clearly delineate which findings are likely context-specific (central Virginia, U.S. healthcare system) versus potentially transferable to other settings.

Final Recommendation

This manuscript addresses a highly relevant clinical and public-health issue and has the potential to make a meaningful contribution to the literature. However, conceptual clarification, strengthened methodological transparency, and more cautious interpretation are required before the work is acceptable.

**********

what does this mean?). If published, this will include your full peer review and any attached files.

If you choose “no”, your identity will remain anonymous but your review may still be made public.

**Do you want your identity to be public for this peer review?**  For information about this choice, including consent withdrawal, please see our Privacy Policy

Reviewer #1: No

**********

[NOTE: If reviewer comments were submitted as an attachment file, they will be attached to this email and accessible via the submission site. Please log into your account, locate the manuscript record, and check for the action link "View Attachments". If this link does not appear, there are no attachment files.]

To ensure your figures meet our technical requirements, please review our figure guidelines: https://journals.plos.org/plosone/s/figures

You may also use PLOS’s free figure tool, NAAS, to help you prepare publication quality figures: https://journals.plos.org/plosone/s/figures#loc-tools-for-figure-preparation.

NAAS will assess whether your figures meet our technical requirements by comparing each figure against our figure specifications.

# Author comment: Author response to Decision Letter 1

**DOI:** 10.1371/journal.pone.0355952.r002

9 Jul 2026

Please see attached Response to Reviewers document for detailed responses to all reviewer and editor comments.

Attachment Submitted filename: Response to Reviewers (2).docx

# Editor report: Decision Letter 1

**DOI:** 10.1371/journal.pone.0355952.r003

**Authors:** Tanja Grubić Kezele

29 Jul 2026

Lived Experiences and Perspectives of Persons with Conditions Marked by Autonomic Dysfunction

PONE-D-25-59300R1

Dear Dr. Shah,

We’re pleased to inform you that your manuscript has been judged scientifically suitable for publication and will be formally accepted for publication once it meets all outstanding technical requirements.

Within one week, you’ll receive an e-mail detailing the required amendments. When these have been addressed, you’ll receive a formal acceptance letter and your manuscript will be scheduled for publication.

An invoice will be generated when your article is formally accepted. Please note, if your institution has a publishing partnership with PLOS and your article meets the relevant criteria, all or part of your publication costs will be covered. Please make sure your user information is up-to-date by logging into Editorial Manager at Editorial Manager® and clicking the ‘Update My Information' link at the top of the page. For questions related to billing, please contact billing support.

If your institution or institutions have a press office, please notify them about your upcoming paper to help maximize its impact. If they’ll be preparing press materials, please inform our press team as soon as possible -- no later than 48 hours after receiving the formal acceptance. Your manuscript will remain under strict press embargo until 2 pm Eastern Time on the date of publication. For more information, please contact onepress@plos.org.

Kind regards,

Tanja Grubić Kezele, Ph.D., M.D.

Academic Editor

PLOS One

Additional Editor Comments (optional):

Reviewers' comments:

# Editor report: Acceptance letter

**DOI:** 10.1371/journal.pone.0355952.r004

**Authors:** Tanja Grubić Kezele

PONE-D-25-59300R1

PLOS One

Dear Dr. Shah,

I'm pleased to inform you that your manuscript has been deemed suitable for publication in PLOS One. Congratulations! Your manuscript is now being handed over to our production team.

At this stage, our production department will prepare your paper for publication. This includes ensuring the following:

* All references, tables, and figures are properly cited

* All relevant supporting information is included in the manuscript submission,

* There are no issues that prevent the paper from being properly typeset

You will receive further instructions from the production team, including instructions on how to review your proof when it is ready. Please keep in mind that we are working through a large volume of accepted articles, so please give us a few days to review your paper and let you know the next and final steps.

Lastly, if your institution or institutions have a press office, please let them know about your upcoming paper now to help maximize its impact. If they'll be preparing press materials, please inform our press team within the next 48 hours. Your manuscript will remain under strict press embargo until 2 pm Eastern Time on the date of publication. For more information, please contact onepress@plos.org.

You will receive an invoice from PLOS for your publication fee after your manuscript has reached the completed accept phase. If you receive an email requesting payment before acceptance or for any other service, this may be a phishing scheme. Learn how to identify phishing emails and protect your accounts at https://explore.plos.org/phishing.

If we can help with anything else, please email us at customercare@plos.org.

Thank you for submitting your work to PLOS ONE and supporting open access.

Kind regards,

PLOS ONE Editorial Office Staff

on behalf of

Prof. dr. Tanja Grubić Kezele

Academic Editor

PLOS One
